# Electrochemical Deposition of Cu/Cu_2_O Nanostructures for Enhanced CO_2_ Reduction Reaction

**DOI:** 10.1021/acsomega.5c08893

**Published:** 2025-11-04

**Authors:** Rabin Dahal, Kiran Subedi, Gayani Pathiraja, Bishnu Prasad Bastakoti

**Affiliations:** † Department of Chemistry, North Carolina A&T State University, 1601 E Market St, Greensboro, North Carolina 27411, United States; ‡ College of Agriculture and Environmental Sciences, North Carolina A&T State University, 1601 E Market St, Greensboro, North Carolina 27411, United States; § Department of Nanoscience, Joint School of Nanoscience & Nanoengineering, 14616 University of North Carolina at Greensboro, Greensboro, North Carolina 27401, United States

## Abstract

We synthesized a dendritic-like Cu/Cu_2_O nanocomposite using a facile electrochemical deposition method on functionalized carbon cloth. The morphology of the electrocatalyst was characterized by using techniques such as scanning electron microscopy and transmission electron microscopy, which revealed the formation of a dendritic nanocomposite. The phase and surface composition were analyzed by X-ray diffraction and X-ray photoelectron spectroscopy. Similarly, electrochemical properties were also studied using cyclic voltammetry and linear sweep voltammetry. The results demonstrated that the electrocatalyst exhibited excellent performance in the electrochemical reduction of CO_2_ to ethene. Specifically, the Faradaic efficiency (FE) at a shorter deposition time (250 s) reached 85.63%, with 62.08% for C2 products, at a current density of 63.57 mA/cm^2^ at −0.97 V versus RHE in H-type cells. The electrochemically active surface area was calculated to be 41.95 cm^2^. The enhanced catalytic activity was attributed to the synergistic effects between Cu^+^ and Cu^0^, which increased the number of active sites, facilitated faster electron transfer, and improved CO_2_ adsorption capacity.

## Graphical abstract

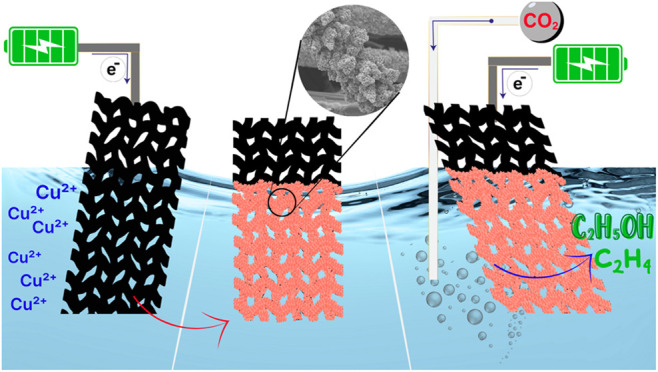

## 1 Introduction

The electrochemical CO_2_ reduction reaction (CO_2_RR) offers a promising approach to mitigate CO_2_ emissions and utilize renewable energy by converting CO_2_ and water into valuable carbon-based fuels and chemicals. However, several challenges, including low conversion rates, high energy consumption, poor selectivity, and competition from the hydrogen evolution reaction (HER), hinder its practical application.^1,2^ Developing an efficient electrocatalyst to address these limitations is crucial. Copper (Cu) is a promising candidate for an electrocatalyst for CO_2_ conversion due to its ability to generate various carbon-based products. The slow and complex reaction pathways involved in C2 product formation lead to reduced selectivity in copper-based catalysts. To address those issues, the metal/metal oxide heterojunction is most widely accepted as compared to metal or metal oxide due to its effective surface charge transfer, which involves shifting electrons between the metal and its ions at different active sites.^3,4^ Several strategies have been employed to enhance the Cu/Cu_2_O heterojunction, including surface modification,^5^ selective deposition,^6^ halogen adsorption,^7^ and doping, among others. These modifications are achieved using hydrothermal treatment,^8,9^ plasma treatment,^10^ in situ reduction process,^11,12^ and annealing.^2^ Tandava et al. fabricated an oxide-derived copper heterostructure supported by carbon using a hydrothermal method, achieving C_2_H_4_ enhancements of 46% FE at −1.6 V vs RHE.^13^ Gao et al. used CNT/bacterial cellulose (BC) nano composite film to prepare Cu–Cu_*x*_O/CNT/BC nanocomposite electrode through in situ chemical reduction for efficient electroreduction of CO_2_ into C_2_H_4_, achieving 30% FE at −1.5 V vs RHE.^14^ Jiang et al. introduced iodine to promote a dynamic Cu/Cu^+^ interface during electrochemical CO_2_ reduction, achieving the FE of 70% for the C2 product.^15^ Furthermore, Li et al. enhanced the selectivity toward C_2_H_4_ over CH_4_ using a Cu/Cu_2_O composite catalyst on N-doped carbon. The FE was found to be 40% toward C_2_H_4_.^2^

Although these methods are widely used, they still have some limitations, including multiple steps, higher temperatures, variable morphological structures, and the need for a capping agent. To address all those limitations, electrochemical deposition techniques have emerged as widely proven and superior alternatives for synthesizing the electrocatalyst.^16,17^ For example, Mech et al. studied the electrochemical and photoelectrochemical conversion of CO_2_ using an electrodeposited Cu–Cu_2_O catalyst, where the FE of C_2_H_4_ was 8.99%.^18^ Shao et al. used dendritic Cu/Cu_2_O synthesized via electrodeposition in CO_2_-saturated KHCO_3_ solution for effective electrocatalytic reduction of CO_2_ into C2 products, where the FE of C_2_H_4_ was found to be 51.42%.^19^ It has been demonstrated that Cu catalysts produced from oxides can enhance the production of C2 products. This enhancement was ascribed by some investigations into the synergistic action of Cu^0^ and Cu^+^ sites in supporting the C–C coupling mechanism.^2^

Herein, an easy and effective method was employed to deposit a Cu/Cu_2_O nanocomposite on carbon cloth, thereby enhancing the electrochemical performance and efficiency in C_2_H_4_ formation. Carbon cloth is used to prepare a flexible electrode that successfully anchors the nanoparticles during electrodeposition for effective charge transfer between the electrode and solution without participating in the electrochemical reduction mechanism and also eliminates the addition of extra chemicals for the capping agent. Similarly, forming a Cu/Cu_2_O nanocomposite also provides a synergistic effect, facilitating C–C coupling during electrochemical CO_2_ reduction through the formation of complex grain boundaries.

## 2 Experimental Section

### 2.1 Materials

Copper sulfate pentahydrate was purchased from Lab Chem, USA. Carbon cloth was purchased from Fuel Cell, USA. Sulfuric acid (95–98%) was purchased from Fisher Chemical, USA. Nitric acid (68–70%) was purchased from Thermo Scientific, USA. Potassium hydroxide (99.98%) was purchased from Acros Organic, Belgium. Deuterium oxide (99.8%) for NMR analysis was purchased from Sigma-Aldrich, Switzerland. 2,2-Dimethyl-2-silapentane-5-sulfonic acid sodium salt (DSS) was purchased from Norell Inc., USA. The chemicals used were of analytical grade and used without further purification.

### 2.2 Synthesis of Catalyst

Before catalyst preparation, the commercial hydrophobic carbon cloth was cut into the dimensions of 1.5 cm × 1 cm and was functionalized by heating in a mixture of nitric acid and sulfuric acid (1:3 v/v) at 60 °C for 2 h. The functionalized carbon cloth (FCC) was cooled and washed with DI water. The prepared FCC was used as a substrate for electrodeposition. A 100 mL solution of 0.1 M acidified CuSO_4_ was prepared by adding 50 μL commercial concentrated H_2_SO_4_ (18 M). Fifteen mL of solution was taken in a beaker, where FCC, a reference electrode (Ag/AgCl), and Pt were dipped as the counter electrode. The constant potential of −0.4 V vs Ag/AgCl was applied for different time intervals (250 s, 500 s, and 1000 s) during electrodeposition. The electrodeposited FCC was dried in an oven at 50 °C for 2 h after washing with DI.

### 2.3 Characterization

X-ray diffraction (XRD) patterns were recorded using Rigaku Miniflex 600 (2θ: 10–80°, step: 0.02, and continuous: 1°/min). A field emission scanning electron microscope (FESEM, JEOL, JSM-IT800) was used to determine the morphology of the samples. Furthermore, the morphology and crystallinity of Cu/Cu_2_O nanostructures deposited on a carbon cloth substrate were also investigated using a JEOL 2100PLUS high-resolution transmission electron microscope (HR-TEM) operated at an accelerating voltage of 200 kV. X-ray photoelectron spectroscopy (XPS) was used to study the elemental composition and chemical state using a Thermo Scientific Escalab XI+ (200 eV and Al Kα). The catalyst synthesis and electrochemical measurements were performed using a CH instrument. Gas Chromatography (GC) (SRI 8610C) was used to study the gaseous product. The liquid products were analyzed using nuclear magnetic resonance (^1^H NMR) (Bruker Ascend 400) and an Agilent 7890B GC system coupled with a 5977B MSD.

### 2.4 Electrochemical Measurements

The electrochemical measurements were performed in an H-type cell with a three-electrode system. The H-type cell consists of platinum (as a counter electrode), an Ag/AgCl electrode (as a reference electrode stored in KCl solution), and FCC with Cu/Cu_2_O (as a working electrode) in 1 M KOH solution. Cyclic voltammetry was measured at scan rates ranging from 60 mV/s to 120 mV/s. Electrochemical impedance spectroscopy (EIS) was used to study the electrode’s conductivity over a frequency range of 0.1 Hz to 100,000 Hz. Likewise, linear sweep voltammetry (LSV) was studied between 0 and 0.1 V vs the reversible hydrogen electrode (RHE) at a scan rate of 10 mV/s. The following equation was used for the calculation of RHE

$$E_{\mathrm{RHE}}=E_{\mathrm{Ag}/\mathrm{AgCl}}+0.197+0.059\;\mathrm{pH}$$

where *E*_Ag/AgCl_ represents the potential against the reference electrode and 0.197 denotes the standard potential of Ag/AgCl at 25 °C.

A 100 mL H-type cell was used for electrochemical measurements. The Nafion 117 membrane was treated with 0.1 M H_2_SO_4_ and deionized water before being used as a separator for two compartments (anodic and cathodic). 35 mL of electrolyte was used in both the compartments. Pure CO_2_ gas (99.9%) was bubbled in a cathodic compartment for 60 min at 3 sccm with the aid of a mass flow controller (MC-100SCCM-D, Alicat Scientific). The outlet of the cathodic cell compartment was connected to the gas chromatograph (GC). The GC (SRI 8610C) was calibrated with a standard gas mixture. The gaseous products obtained during the reaction were detected by a flame ionization detector (FID). Helium gas with a flow rate of 15 was used as the carrier gas in the GC analysis. The liquid products were analyzed using nuclear magnetic resonance (^1^H NMR) (Bruker Ascend 400) and an Agilent 7890B GC system coupled with a 5977B MSD. The inlet temperature was maintained at 250 °C, and 0.1 μL of sample was injected in split mode (1:28). The oven was initially held at 120 °C for 0.8 min, then ramped at 9 °C/min to 150 °C, where it was held for 1 min. Separation was achieved using an Agilent HP-5MS capillary column. Helium served as the carrier gas at a constant flow rate of 1.0 mL/min. Mass spectra were acquired over a scan range of 20–100 amu, and compound identification was confirmed using the NIST 20 library. The potential of −1.2 V vs RHE was applied for current–time (*i*–*t*) measurements. Gases from the cell were injected into the GC for 400 s, and electrocatalytic CO_2_RR was evaluated.

## 3 Results and Discussion

Figure 1 shows the XRD pattern of electrodeposited FCC at different time intervals. The electrodeposited functionalized carbon cloth displays diffraction peaks at 2θ (*hkl*) of 36.5° (111), 42.67° (200), and 61.6° (220), confirming the formation of cuprite, (Cu_2_O). These peaks align well with the standard diffraction card for a body-centered cubic structure (PDF 005–0667). In addition to these, some other diffraction peaks are observed at 43.66° (111), 50.68° (200), and 74.41° (220), which correspond to metallic copper. According to standard diffraction cards, these peaks align with a face-centered cubic structure (PDF-004–0836). The peak near 26° corresponds to carbon cloth, as shown in Figure S1 (Supporting Information). As the deposition time increases, the peaks of metallic copper become more intense due to the accumulation of more copper on the surface of the FCC. The result showed the formation of both Cu and Cu_2_O nanocomposites. The full-width half-maximum was also calculated, which showed the broadening of the peak by changing the deposition time from 250 s to 500 s. However, an intense peak (less broadening) was observed at 1000 s when the deposition time was increased, due to the increase in Cu concentration over the FCC, which in turn increases the crystallinity. The crystallite size of the peaks was calculated by Scherrer’s formula.^20^ The crystallite sizes calculated were 18.75, 16.80, and 23.76 nm for 250 s, 500 s, and 1000 s of deposited time. The increase in crystallite size is due to the change in growth mechanism in the presence of hydrogen gas evolved at the cathode or electrolyte interface. Initially, the crystallite size is also greater due to the active growth of early formed nuclei under a high driving force. As the deposition continues, the crystallite size decreases due to increased nucleation density, resulting in the formation of many small grains and limited growth space. However, with prolonged deposition time, the crystallite size rises again due to processes such as Ostwald ripening and grain boundary movement, which promote the coalescence of smaller grains into larger ones.^21,22^ The decrease in peak intensity also confirmed the increase in Cu/Cu_2_O deposition over time.

**Figure 1:**
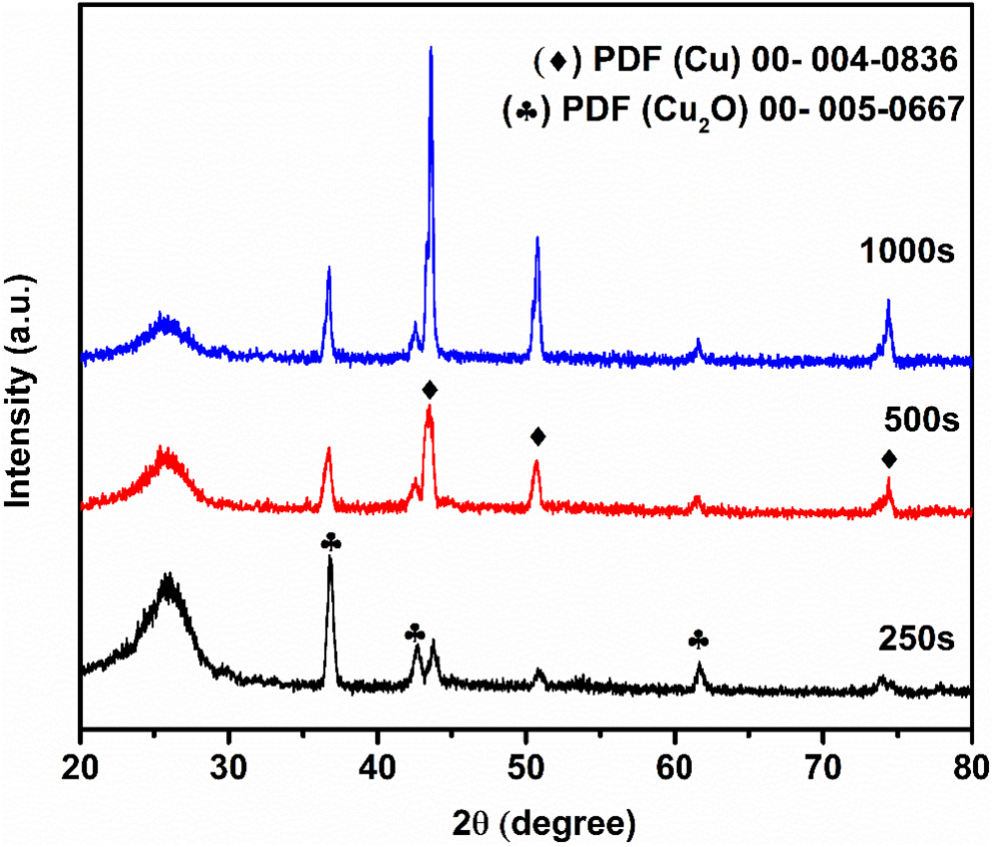
XRD patterns of electrodes deposited at different time intervals of 250 s, 500 s, and 1000 s.

The surface morphology of electrodeposited FCC was investigated using field-emission scanning electron microscopy (FESEM). The FESEM image in Figure 2 shows the formation of the dendrite structure of a Cu/Cu_2_O nanocomposite at the same potential but at different deposition times. Transmission electron microscopy (TEM) analysis was employed to characterize the morphology and crystallinity of Cu/Cu_2_O nanocomposites after they were electrodeposited on a carbon cloth substrate. Figure 2(d) displays nanoflower structures and small nanoparticles with a 2–10 nm particle size. At a higher magnification of the expanded region of nanoflowers, high-resolution transmission electron microscopy (HR-TEM) revealed the lattice spacing of Cu_2_O to be 0.25 nm, corresponding to the (111) crystal plane, as shown in Figure 2(e),(g). Similarly, the crystal lattice spacings of the expanded region of the nanoparticles are found to be 0.21 nm, oriented in the (111) direction, as shown in Figure 2(f),2(h). Additionally, we observed the presence of carbon cloth with Cu/Cu_2_O nanostructures in the TEM image. The selected area electron diffraction (SAED) pattern supports the crystallinity of these nanostructures, whereas Figure 2(i) shows (111) reflections from both Cu_2_O and Cu.^23^

**Figure 2:**
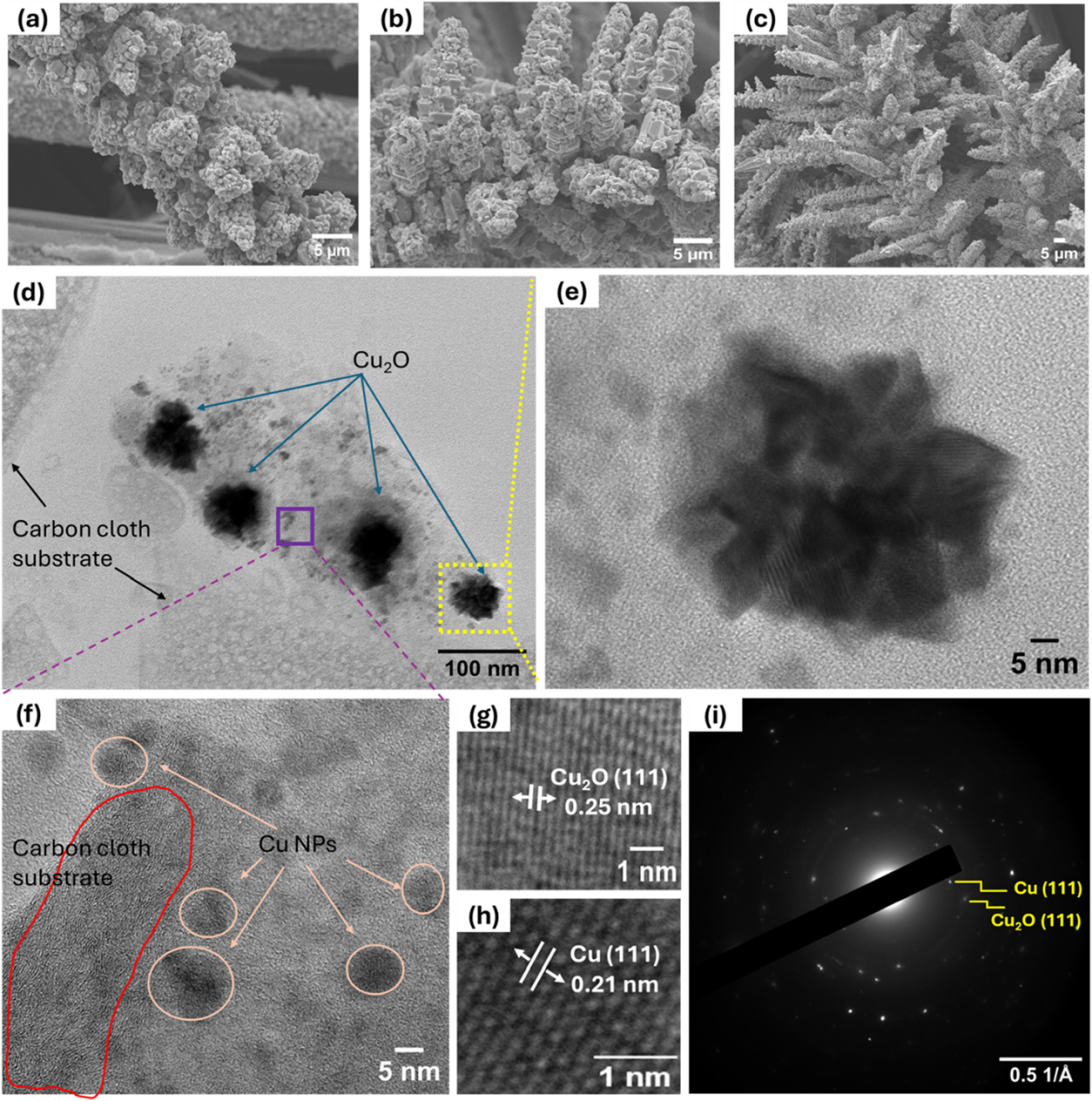
SEM images of electrodeposited Cu/Cu_2_O on FCC at different deposition times (a) 250 s, (b) 500 s, and (c) 1000 s. TEM images of (d) Cu/Cu_2_O nanoparticles on carbon cloth substrate, (e) HR-TEM of an expanded Cu_2_O nanoflowers region (yellow highlight), (f) HR-TEM of an expanded Cu nanoparticles region (purple highlight), (g) the respective lattice spacings of Cu_2_O­(111), (h) Cu(111) crystal planes and (i) corresponding selected area electron diffraction pattern of TEM image (d).

The survey scan in Figure 3(a) shows the presence of Cu 2p, O 1s, and C 1s spectra, characterized by the binding energies at 932, 530, and 284 eV, respectively. The high-resolution spectra of Cu 2p are plotted in Figure 3(b), where the peaks at 932.55 eV (Cu 2p_3/2_) and 952.34 eV (Cu 2p_1/2_) are related to the Cu^+^ or Cu^0^ species which were also observed in 500 s and 1000 s electrodeposited FCC shown in Figures S2­(b) and S3­(b) (Supporting Information). Furthermore, the peak at the binding energy level of 943.56 eV is associated with the satellite peak of Cu^+^. In addition to those peaks, the 934.96 and 954.37 eV peaks confirm the presence of Cu^2+^ species on the surface. This is due to the oxidation of Cu_2_O to CuO by oxygen in the air during the drying process. Similarly, Figure 3(c) displays the deconvoluted O 1s spectra. The peak at 530.45 eV confirms the presence of lattice oxygen, whereas the peak at the binding energy of 531.99 eV is attributed to the surface hydroxyl.^24–28^ The high-resolution XPS spectrum of C 1s was deconvoluted as shown in Figure 3(d). The peak at 284.63 eV is attributed to C–C bonds, 285.32 eV is attributed to C–O bonds, and 288.19 eV is attributed to C=O bonds.^29–31^ Similar results were observed in the electrodeposited FCC at 500 s and 1000 s, as shown in Figures S2 and S3 (Supporting Information).

**Figure 3:**
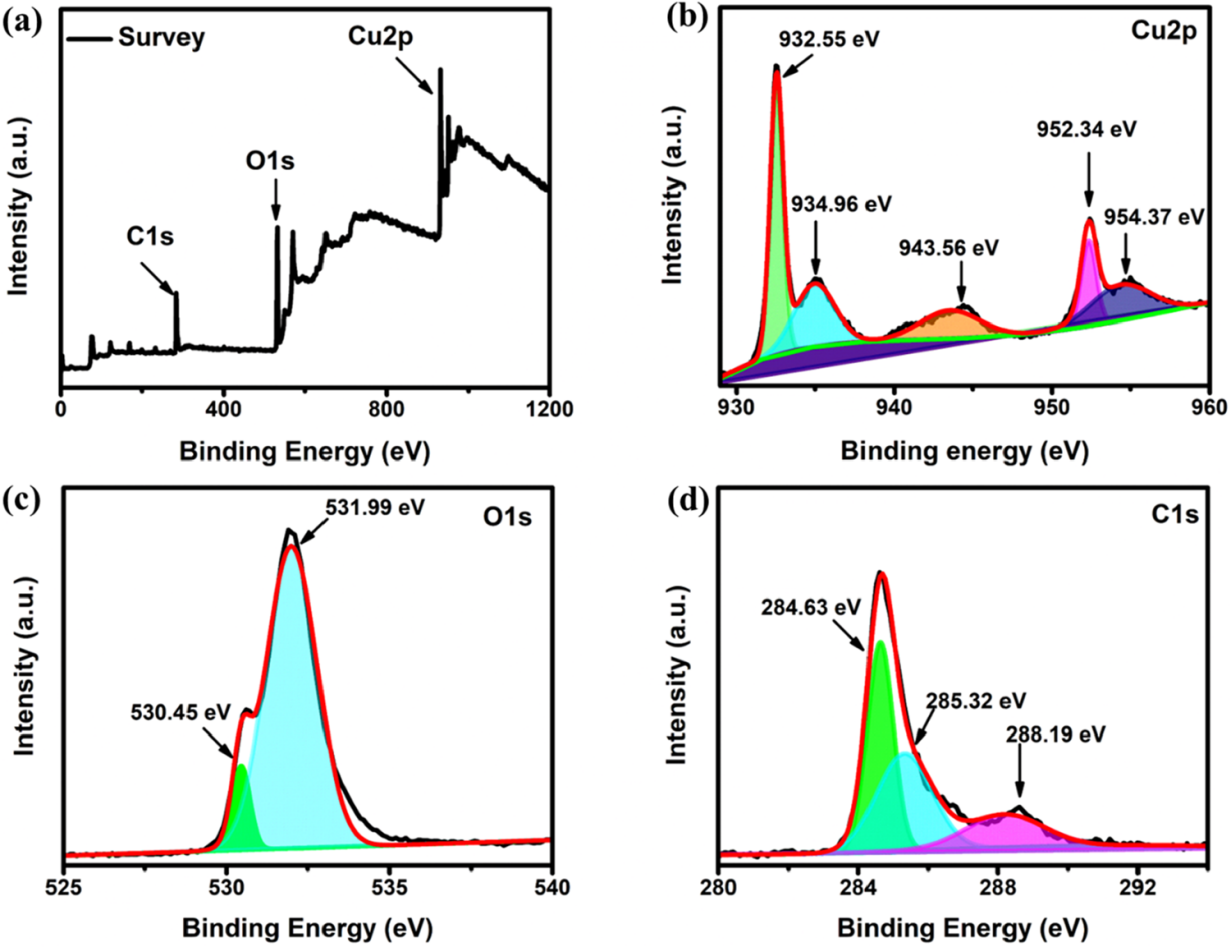
XPS image of electrodeposited FCC at 250 s, (a) Survey scan, (b) Cu 2p spectra, (c) O 1s spectra, and (d) C 1s spectra.

The electrochemical measurements of electrodeposited functionalized carbon cloth were performed in a 3-electrode system in an H-type cell. The cyclic voltammetry profile shown in Figure 4(a) for electrodeposited FCC is recorded between −0.8 and 0.8 V with a scan rate of 100 mV/s. The peak (I) observed between 0.0 and 0.3 V vs Ag/AgCl indicates the oxidation of Cu to Cu_2_O, and the peak (II) between −0.3 V and −0.5 V corresponds to the reduction of Cu_2_O to Cu.^32^ These obtained peaks confirm the redox reaction at the surface of FCC. Among the three samples, 250 s electrodeposited FCC showed the highest current values, confirming the fast electron transfer between Cu/Cu_2_O and FCC, which is attributed to the excellent electrochemical performance.^3^ Similarly, the electrochemically active surface area was calculated from nonfaradaic current regions plotted against different scan rates, as shown in Figure S4 (Supporting Information).^33^ The results show a higher electrochemically active surface area for the 250 s electrodeposited FCC (41.95 cm^2^) compared to the 500 s (18.49 cm^2^) and 1000 s (18.81 cm^2^) electrodes. Additionally, more active sites are present in 250 s electrodeposited catalysts due to less agglomeration, as confirmed by FESEM and HRTEM, which contributes to electrochemical performance.

**Figure 4:**
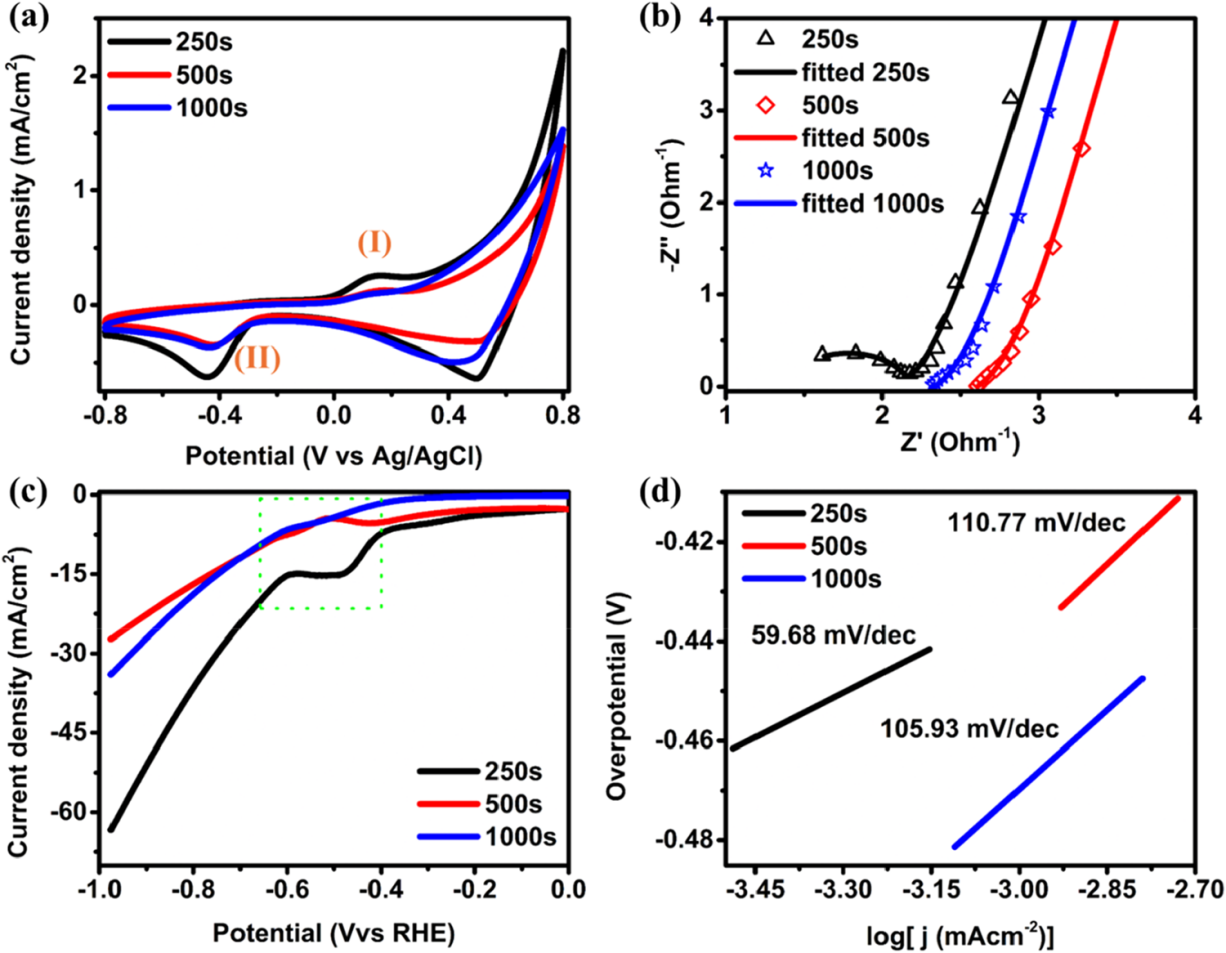
(a) CV comparison curve, (b) EIS comparison curve, (c) LSV curve, and (d) Tafel slope of electrodeposited FCC at different time intervals.

The electrochemical impedance spectroscopy (EIS) of the electrodeposited samples was also investigated to explore the electrocatalytic activity of CO_2_ reduction, as shown in Figure 4(b). EIS is split into two components: the imaginary part (*Z*″ or *Z*_imag_), represented on the *y*-axis, and the real part (*Z*′ or *Z*_real_), displayed on the *x*-axis. This forms a Nyquist plot, where each point on the graph denotes the impedance at a specific frequency, with the imaginary part (*Z*″) being negative.^34^ Compared with other samples, the semicircle obtained at 250 s indicated improved electron transfer efficiency. The equivalent circuit diagram is shown in Figure S5 (Supporting Information). The solution resistance (*R*_1_) of 250 s is 1.41 Ω, which is lower compared to 500 s (2.60 Ω) and 1000 s (2.33 Ω) electrodeposited FCC. Similarly, the charge transfer resistance of the 250 s catalyst (*R*_2_ = 0.70 Ω) is also low compared to the 500 s (*R*_2_ = 5.11 Ω) and 1000 s (*R*_2_ = 3.78 Ω) catalysts, as shown in Table S1. The lower the solution and charge transfer resistance, the more efficient the electron transfer between the solution and the catalyst, which enhances electrode conductivity for effective electrochemical CO_2_ reduction. Figure 4(c) shows the LSV curve of different electrodeposited FCC recorded at 10 mV/s in the applied potential range between 0 V to −1 V (vs RHE). The hump region between −0.6 V and −0.4 V indicates the reduction of copper­(I) oxide to copper, which also supports the reduction process occurring at the electrode, as shown in the CV graph. The 250 s electrodeposited FCC showed the highest current density as compared to 500 s and 1000 s. Notably, the maximum potential value of 63.57 mA/cm^2^ was achieved at −0.97 V (vs RHE). The increase in current density is due to faster electron transport.^35^ This result also supports the EIS analysis. Similarly, the Tafel plot was used to study the reaction kinetics of an electrodeposited catalyst, as shown in Figure 4(d). Linear fitting of the Tafel plot was performed, revealing that the 250 s electrodeposited FCC exhibited the lowest overpotential versus Ag/AgCl compared to other electrodeposited catalysts. The lower overpotential indicated a lower energy barrier for CO_2_ adsorption and activation. This result suggests that 250 electrodeposited samples possess a more active site and enhanced surface area, which facilitates faster and more effective charge transfer.^36^ This result also aligns with the result obtained from EIS and LSV analysis.

An electrochemical CO_2_ reduction was carried out in an H-type cell, where a steady flow of 3 sccm was purged into the cathodic compartment for 400 s, as illustrated in Figure 5(a). The applied potential was −1.2 V versus RHE. The gaseous and liquid products obtained after electrochemical CO_2_ reduction were analyzed using GC, GC-MS, and ^1^H NMR. The faradaic efficiency of all three prepared electrocatalysts was calculated as shown in Figure 5(b) and Supporting Information. The 250 s FCC showed the highest FE of 85.63% compared to the 500 s FCC (57.39%) and 1000 s FCC (75.78%). Among 85.63% FE, 62.08% was for C2 product (C_2_H_2_ and C_2_H_5_OH) in 250 s FCC electrocatalyst. Similarly, for 500 s and 1000 s, the FE for C2 products was 42.25% and 35.03% respectively. Similarly, the electrochemical CO_2_ reduction was also performed at a lower potential of −0.98 V versus RHE, and the result showed better conversion of CO_2_ into product at −1.2 V. The FE of the C2 product was 39.73% for 250s FCC, where no liquid product was detected on ^1^H NMR (Figure S6, Supporting Information). The greater FE of the 250 s FCC is attributed to a higher active surface area and a greater Cu^+^/Cu^0^ interface, as confirmed by ECSA and TEM analysis. The high active surface area provides numerous sites for CO_2_ reduction, which accumulates the intermediate reaction. The increase in the concentration of the C1 intermediate promotes the C–C dimerization due to the Cu^+^/Cu^0^ interface.^37^ This increase in Cu^0^ concentration lowered the efficiency of C2 selectivity as the C–C coupling was hindered by the lower adsorption of the CO intermediate in CO_2_RR. All these analyses show that the increase in Cu_2_O content promotes the C–C coupling, leading to higher C2 production.^2,19,27,38,39^

**Figure 5:**
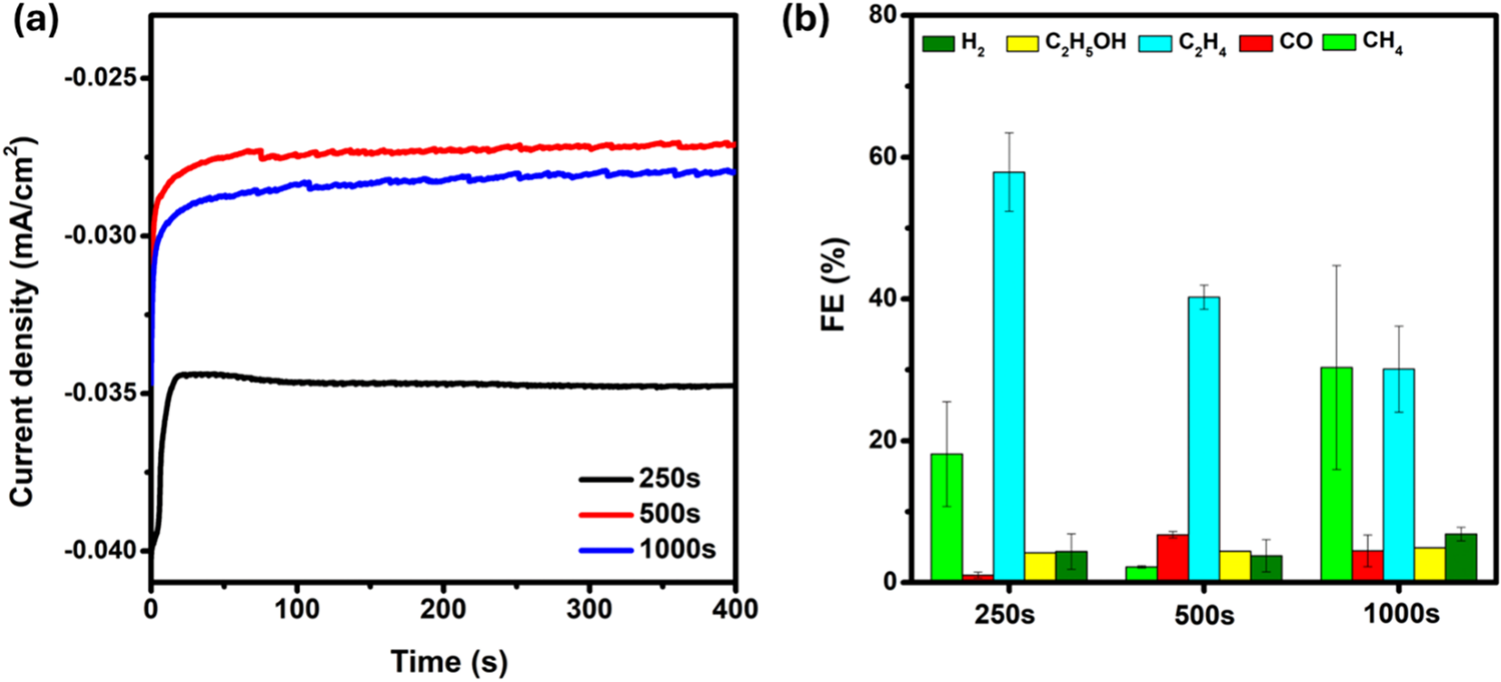
(a) *i*–*t* curves of all the electrodeposited samples at −1.2 V vs RHE for 400 s, and (b) Faradaic efficiency obtained at −1.2 V vs RHE of all the electrodeposited FCC at different deposition times.

The liquid products obtained were investigated by ^1^H NMR and GC-MS analysis, as shown in Figure S7, Supporting Information, where ethanol was confirmed; however, the FE of ethanol was very low compared to ethylene. This result showed ethylene (C_2_H_4_) was more favorable as a C2 product than ethanol (C_2_H_5_OH). This might be due to the lower electrode potential applied, as the activation energy of ethylene is lower than that of ethanol. Furthermore, the applied electrode potential drove the direct hydrogen transfer through surface-coupled hydrogenation to the reaction intermediate due to steric hindrance, which resulted in higher ethylene production.^40,41^

Based on the product obtained during electrochemical reduction, the proposed mechanism for the product formation is as follows. The purged CO_2_ in the solution was first adsorbed and activated in 111 planes of Cu_2_O. The absorption of CO_2_ on the 111 plane was further reduced to *COOH intermediate and *CO intermediate via proton–electron charge transfer. The stability of the *CO adsorption intermediate is the driving factor for the C2 product. The synergetic effect of Cu^+^/Cu^0^ promotes the C–C coupling by dimerizing the two adsorbed molecules of the *CO intermediate. The two adsorbed *CO intermediate forms the *CH–COH intermediate by gaining 4H^+^/e^–^. The *CH–COH is further hydrogenated to generate *C–CH intermediate by losing a water molecule, which finally gives C_2_H_4_ by gaining 3H^+^/e^–^ in the reaction.^42,43^ However, if the *CH–COH intermediate hydrogenates in C species, then there is a possibility of forming *CH_2_–COH, which is a possible pathway for ethanol formation. The generated *CH_2_–COH gets protonated to convert into *CH_2_–CHOH, which finally gives ethanol (CH_3_CH_2_OH) by gaining 2H^+^/e^–^.^44,45^ The formation of methane (CH_4_) is due to the presence of Cu^0^ in the catalyst. As the concentration of Cu^0^ increased, the adsorption of *CO intermediates weakened, increasing methane production. The stability of the 250s FCC for 12 h was also measured, with the result showing that the catalyst remained stable for 12 h at −1.2 V vs RHE, as shown in Figure S8 (Supporting Information). The GC analysis reveals the formation of CO, CH_4_, C_2_H_4_, and H_2_, whereas the ^1^H NMR and GC-MS confirmed the formation of ethanol as a liquid product (Figure S7, Supporting Information). The electrochemical CO_2_ reduction on electrodeposited Cu/Cu_2_O catalysts was compared with different Cu_2_O-based catalysts, as shown in Table S2. After the electrochemical analysis, the electrodeposited catalyst was examined using XRD spectra and FESEM imaging to observe any changes that occurred after the electrochemical measurements. The XRD spectra showed a change in peak intensity at 36.5° and 43.6°, indicating that copper­(I) oxide is reduced to Cu^0^ during electrochemical CO_2_ reduction (Figure S9, Supporting Information). Similarly, FESEM images of the Cu/Cu_2_O electrocatalyst after electrochemical CO_2_ reduction reveal a dendritic structure with alterations and modifications. This demonstrates that the catalyst can be reused for electrochemical CO_2_ reduction (Figure S10, Supporting Information).

## 4 Conclusion

In summary, we synthesized a Cu/Cu_2_O nanocomposite catalyst using electrodeposition on functionalized carbon cloth, varying the deposition times. The electrodeposited catalyst showed improved electrochemical performance, with higher current density and lower overpotential, enabling efficient electrochemical CO_2_ reduction. The produced composite demonstrates high selectivity for C_2_H_4_ because of the presence of Cu^+^ and Cu^0^ active sites. Results indicated that strong adsorption and high coverage of *CO intermediates on the Cu^0^/Cu^+^ sites promote C–C coupling, leading to high C2 selectivity. These findings highlight how surface modification can effectively stabilize catalytically active regions, providing valuable insights for designing reliable and efficient CO_2_RR catalysts.

## Supplementary Material



## Data Availability

The data are available throughout the manuscript and Supporting Files.

## Notes

XRD of carbon cloth; XPS of electrodeposited FCC at 500 s and 1000 s; CV analysis of 250 s, 500 s, and 1000 s electrodeposited samples; Equivalent circuit diagram; Stability of 250 s FCC; NMR analysis and GC-MS analysis of liquid products: XRD of 250 s electrodeposited cloth after electrochemical CO_2_ reduction; FESEM of all the samples after electrochemical CO_2_ reduction. The formula for the quantification of gaseous products and liquid products (PDF)
