# Supplementary material for: Electrochemical Deposition of Cu/Cu2O Nanostructures for Enhanced CO2 Reduction Reaction
Source: ACS Omega. 2025 Nov 4;10(45):54911–8. doi: 10.1021/acsomega.5c08893 (PMC12631392; doi:10.1021/acsomega.5c08893)
Supplement: Supplementary file 1 [file ao5c08893_si_001.pdf]

Supporting Information

Electrochemical Deposition of Cu/Cu_2O Nanostructures for Enhanced CO_2 Reduction Reaction

Rabin Dahal[1], Kiran Subedi[2], Gayani Pathiraja[3] and Bishnu Prasad Bastakoti[1]*

[1]Department of Chemistry, North Carolina A and T State University, 1601 E Market St, Greensboro, NC 27411, USA

[2]College of Agriculture and Environmental Sciences, North Carolina Agricultural and Technical State University, 1601 E Market St, Greensboro, NC 27411, USA

[3]Department of Nanoscience, Joint School of Nanoscience & Nanoengineering, University of North Carolina at Greensboro, Greensboro, North Carolina 27401, USA

bpbastakoti@ncat.edu

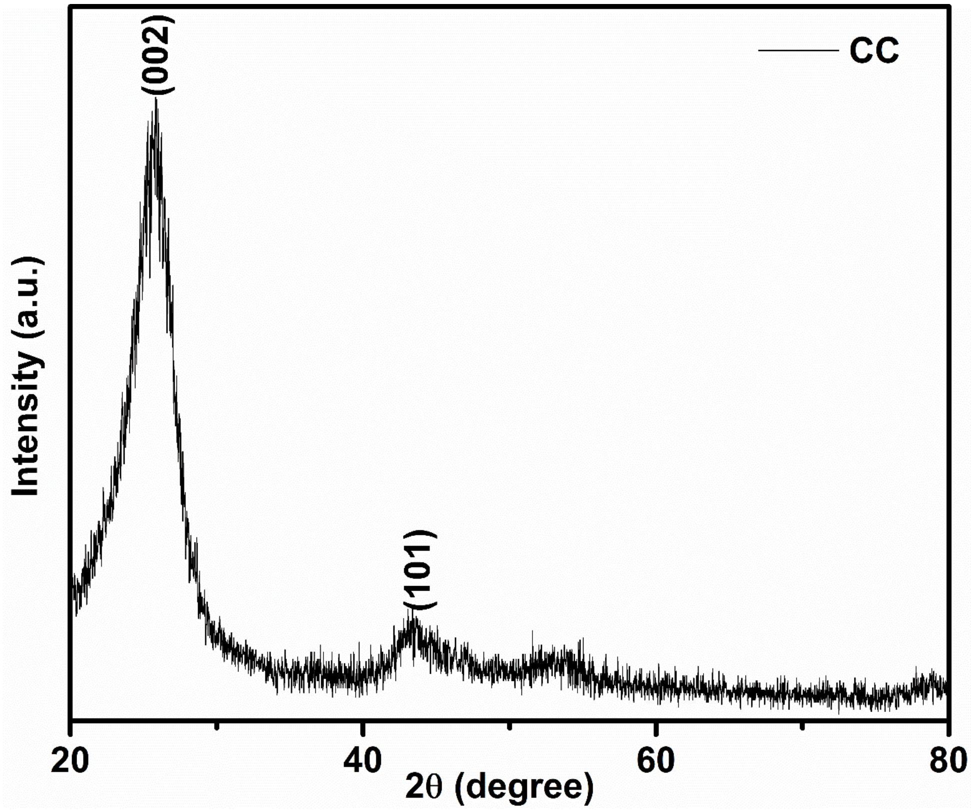

Figure S1. XRD spectra of carbon cloth.

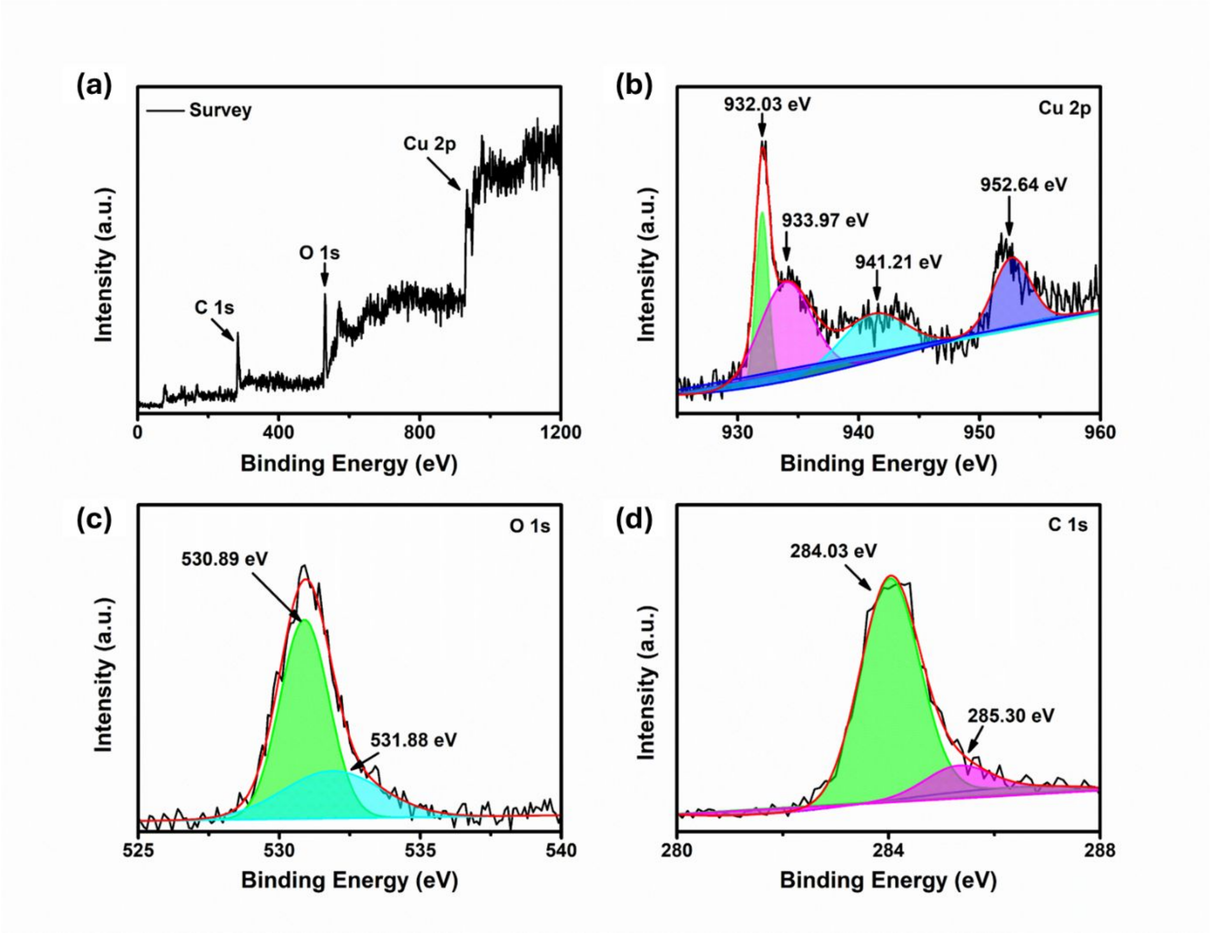

Figure S2: XPS image of electrodeposited FCC at 500s, (a) Survey scan, (b) Cu 2p spectra, (c) O 1s spectra, and (d) C 1s spectra.

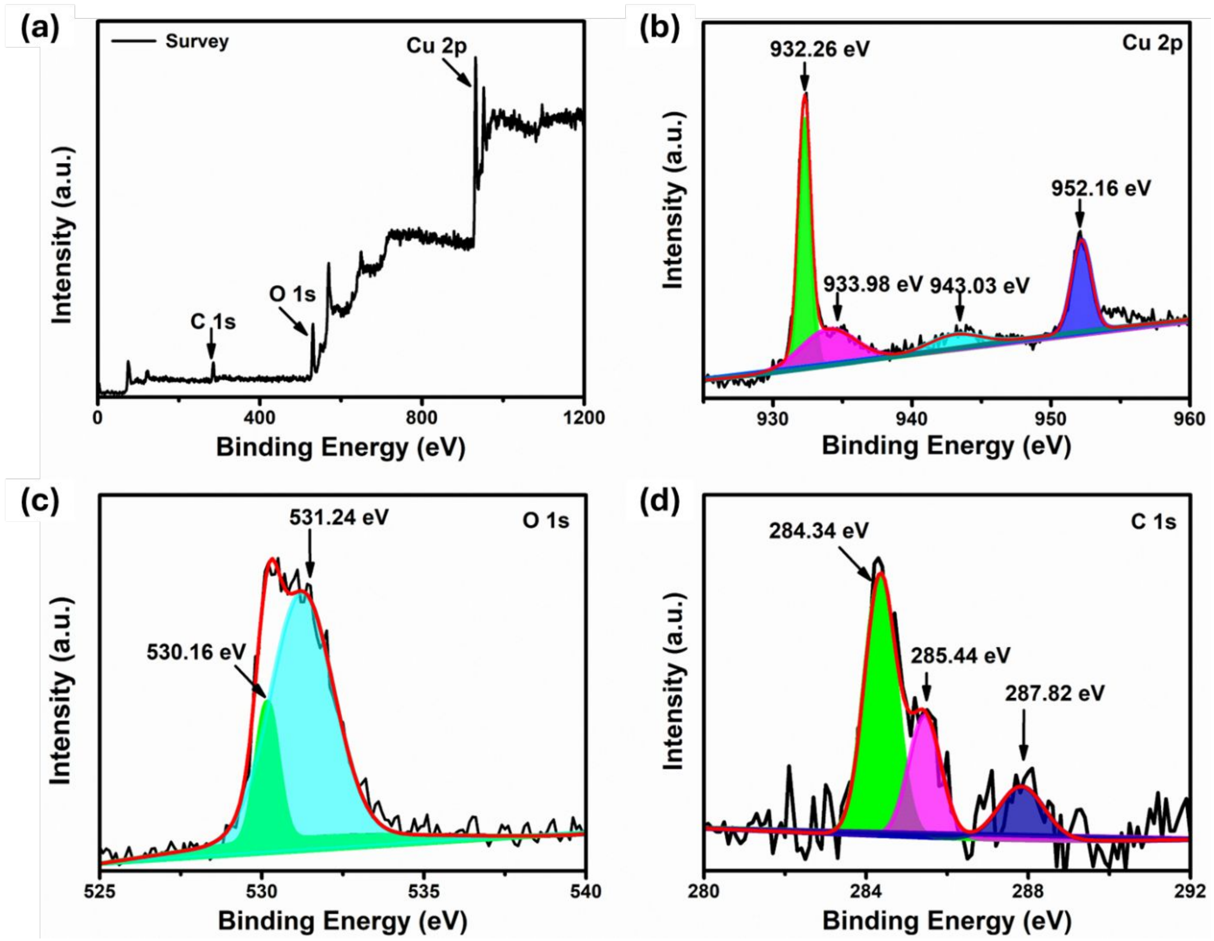

Figure S3: XPS image of electrodeposited FCC at 1000s, (a) Survey scan, (b) Cu 2p spectra, (c) O 1s spectra, and (d) C 1s spectra.

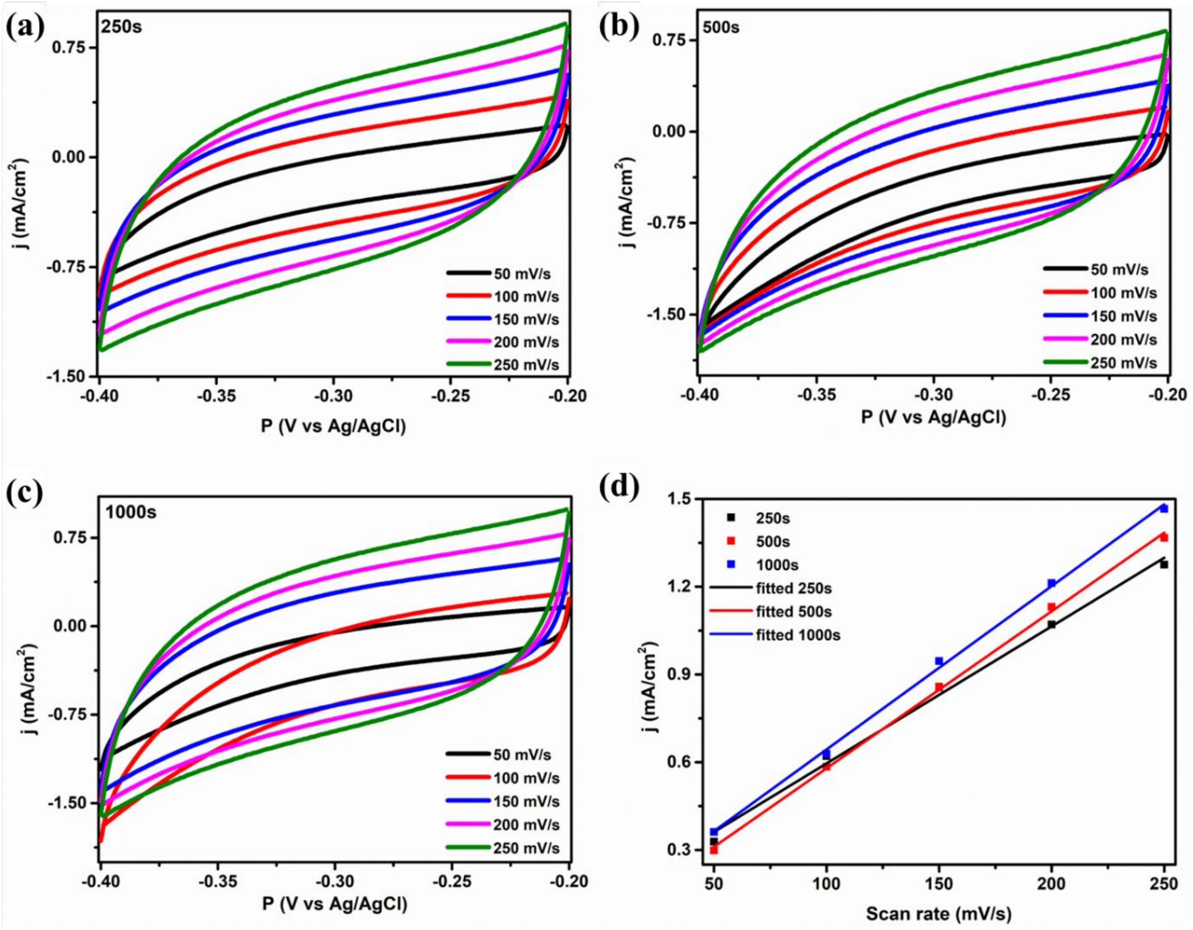

Figure S4. Electrochemical surface area (ECSA) measurements. Cyclic voltammetry (CV) curve of (a) 250s, (b) 500s (c) 1000s, and (d) C_{dl} curve.

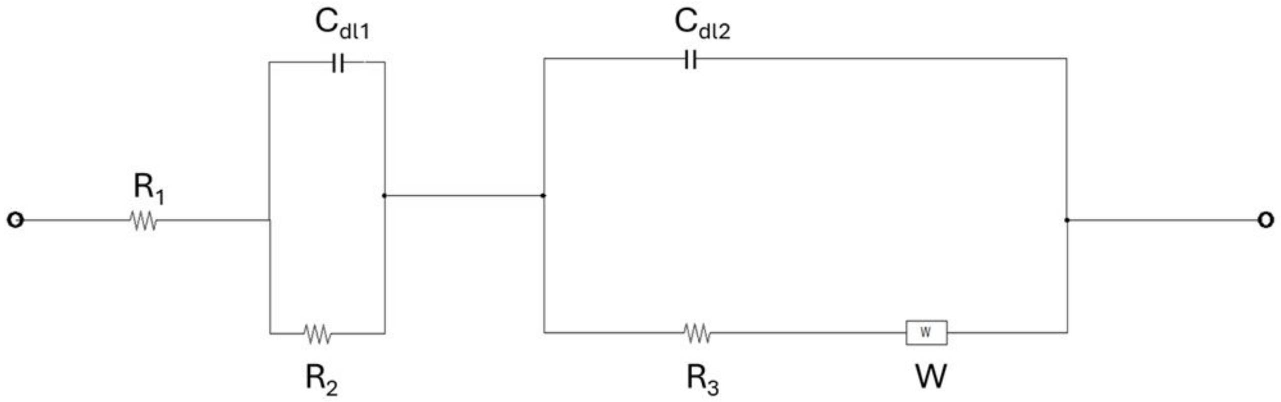

Figure S5. Equivalent circuit diagram used for the fitting process of electrodeposited catalyst.

Table S1: Equivalent series resistance values of samples derived from the EIS analysis.

Samples	R1 (solution resistance) Ω	Warburg impedance coefficient (σ)	R2 (Charge transfer resistance) Ω
250s	1.41	0.00178	0.70
500s	2.60	0.00422	5.11
1000s	2.33	0.00401	3.78

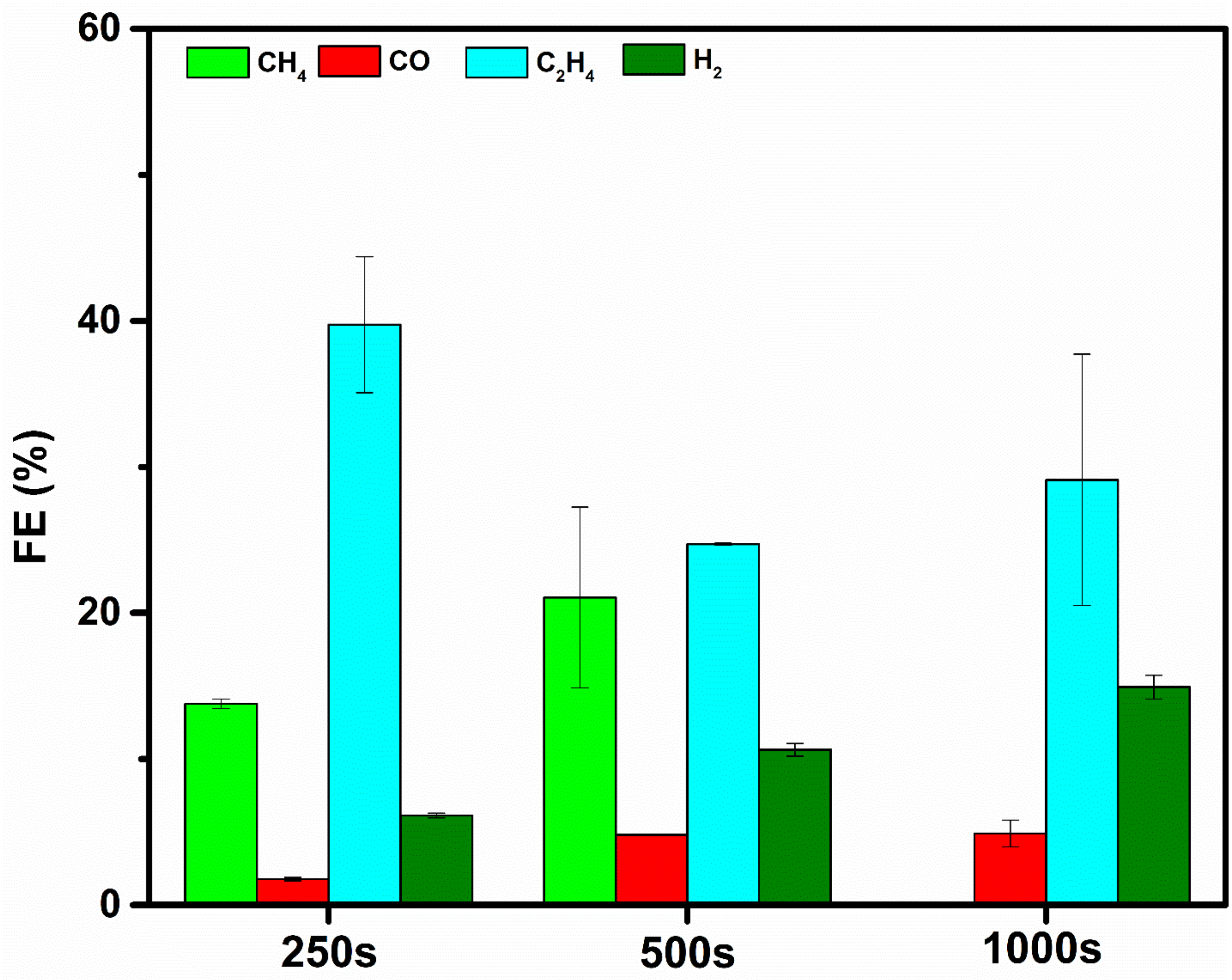

Figure S6. Faradaic efficiency obtained at -0.98 V Vs RHE of all the electrodeposited FCC at different deposition times.

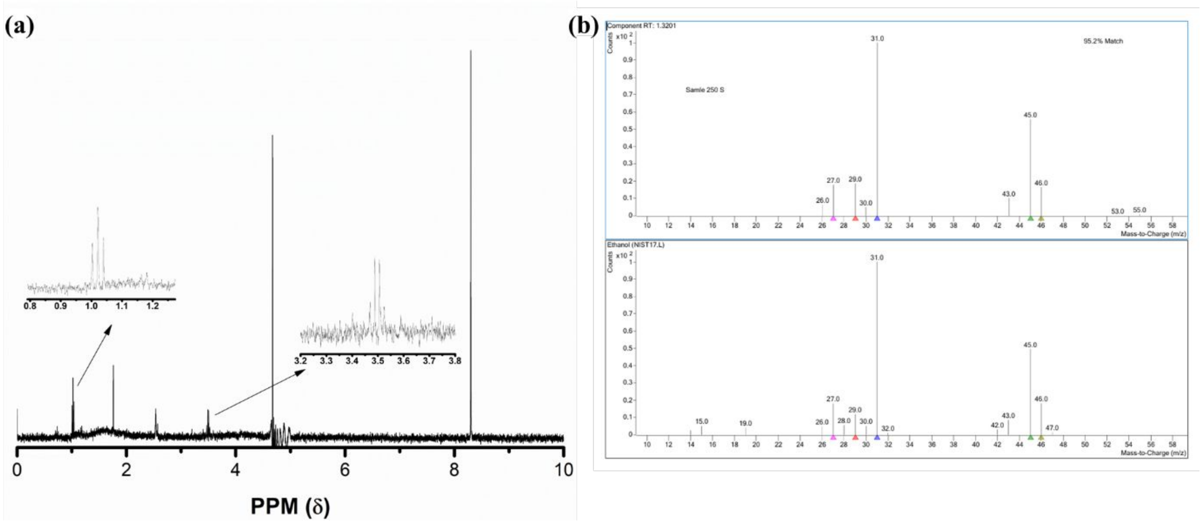

Figure S7. (a) NMR analysis after electrochemical CO_2 reduction and (b) GC-MS comparison spectra with standard samples.

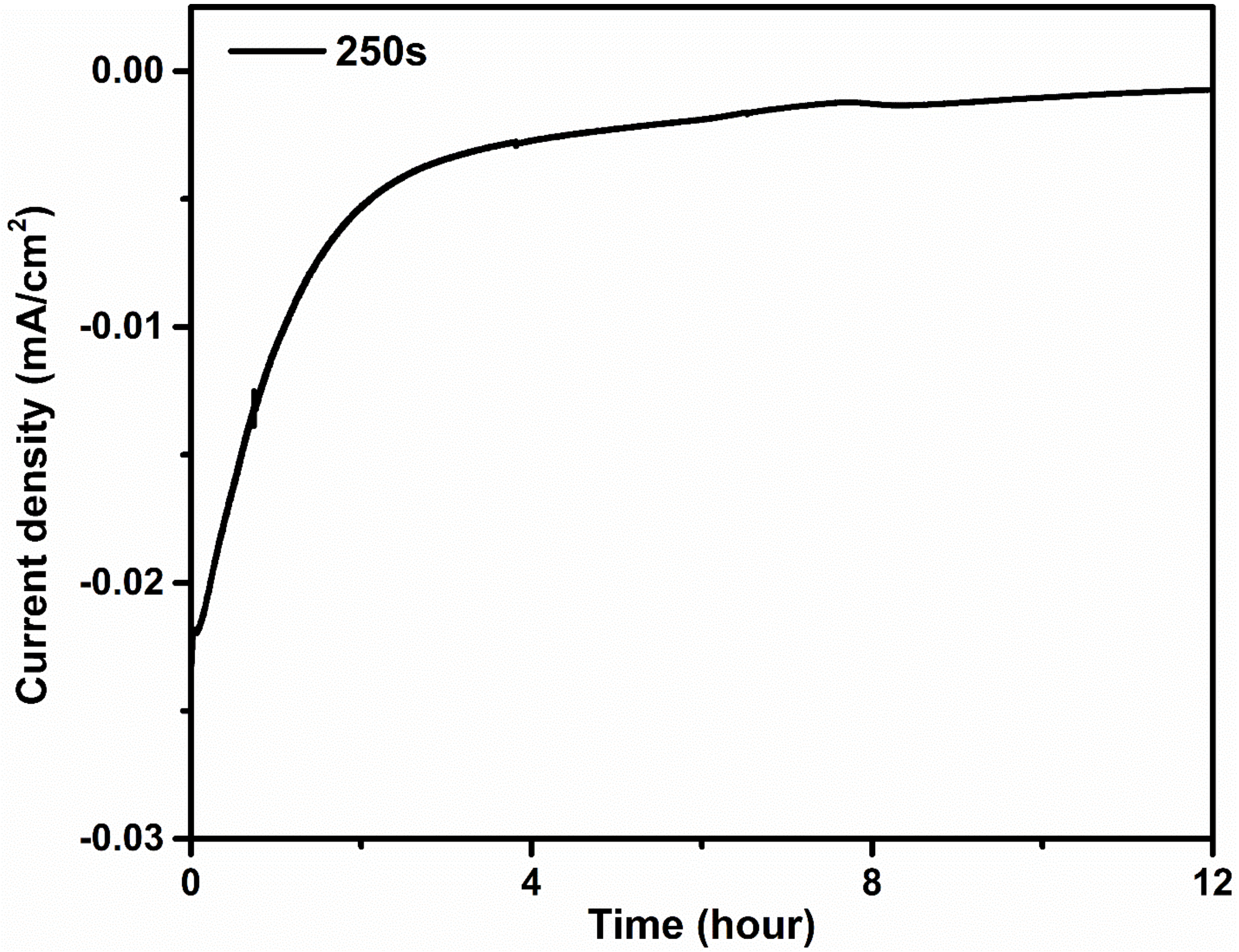

Figure S8. Stability of the 250s electrodeposited FCC at -1.2V Vs RHE for 12 hours.

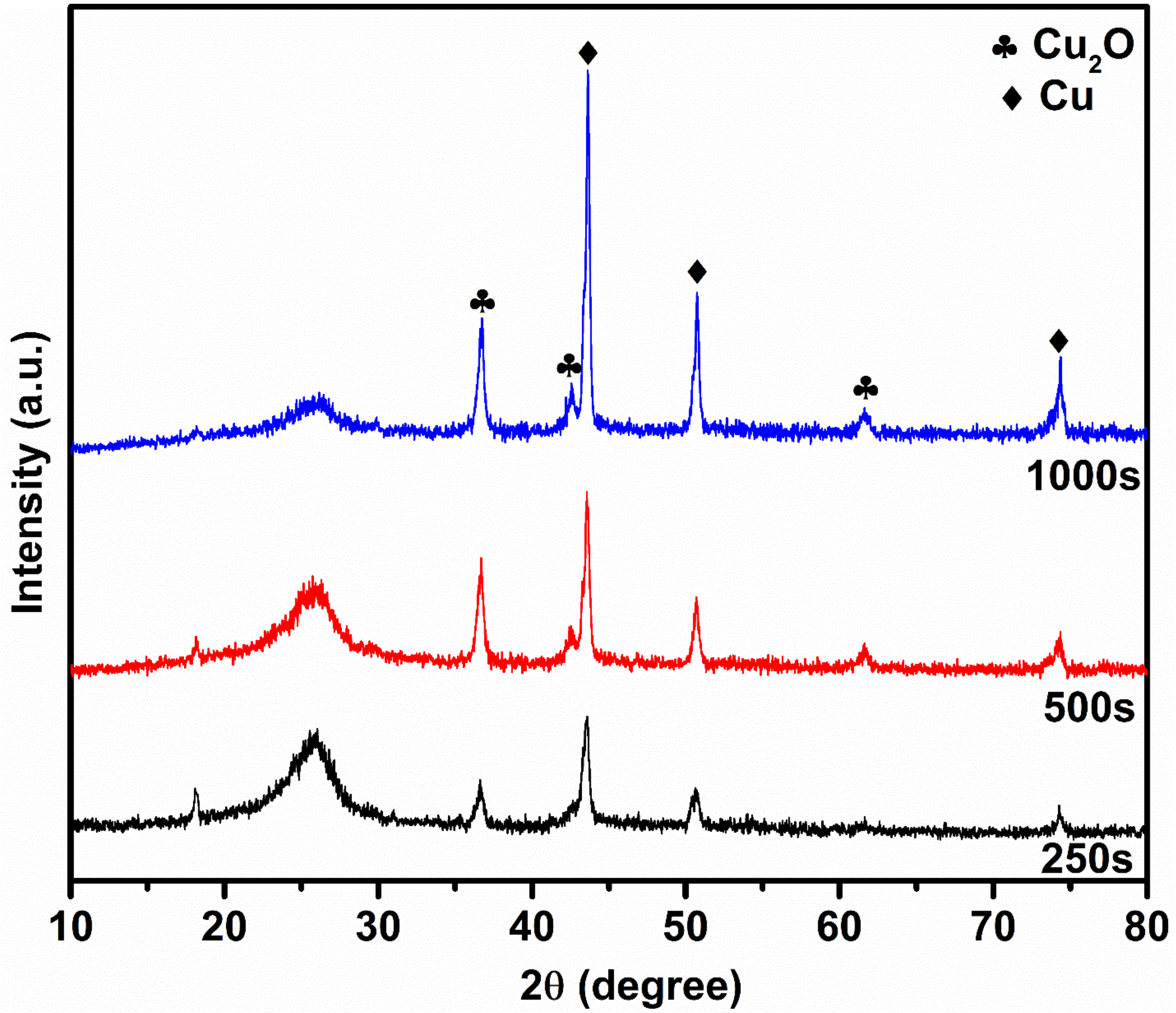

Figure S9. XRD spectra of the electrodeposited catalyst after electrochemical CO_2 reduction.

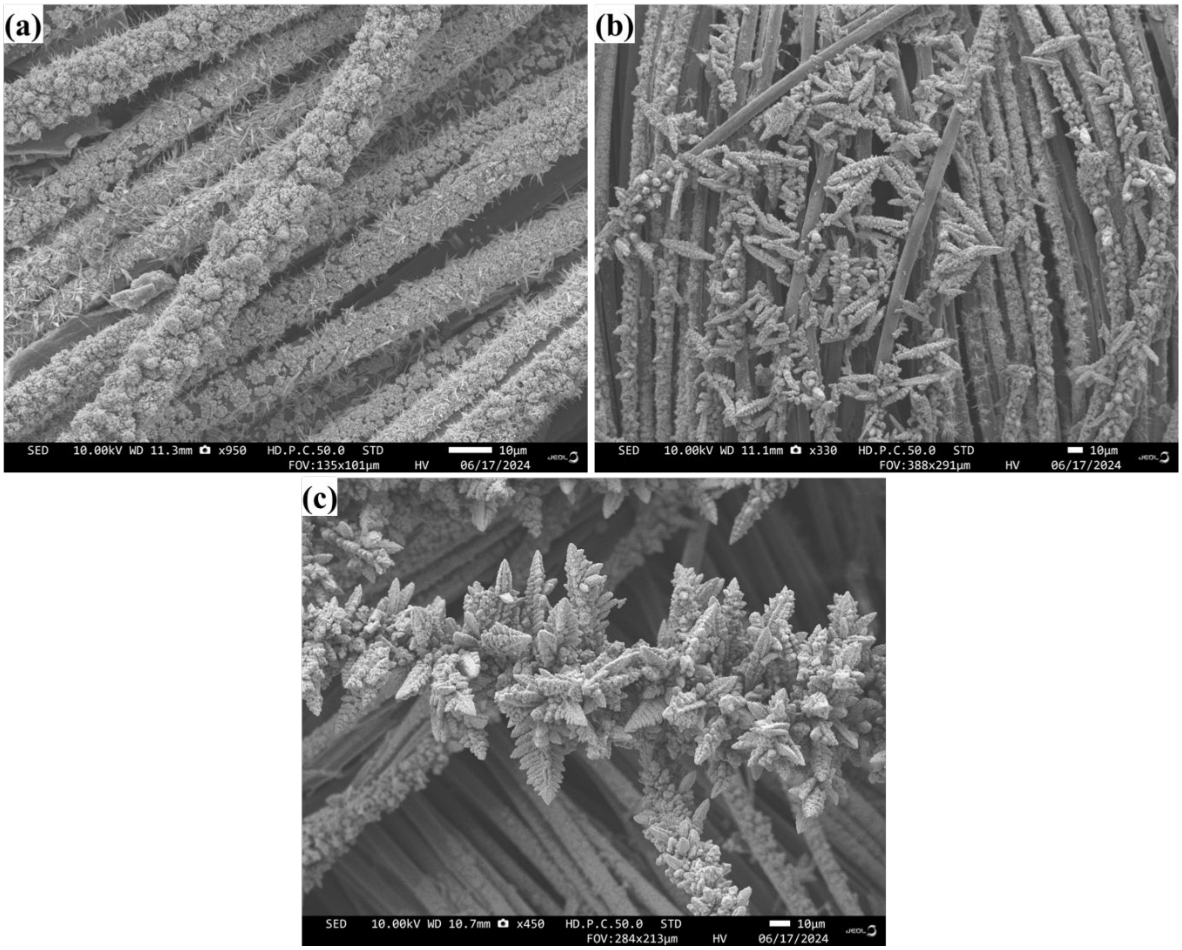

Figure S10. FESEM of electrodeposited Cu/Cu_2O catalyst after electrochemical CO_2 reduction. (a) 250s, (b) 500s and (c) 1000s.

Table S2: Comparison of different Cu_2O catalysts with electrodeposited Cu/Cu_2O.

Catalyst	Synthesis	E Vs RHE	Electrolyte	FE (%)	Ref.
Cu/Cu_2O	Electrodeposition (0.1molL-1CO_2-saturated $KHCO_3$)	-1.9 V	1 molL-1 $KHCO_3$	C_2H_4 (51.42%)	[1]
Cu/Cu_2O aerogel	Chemical synthesis	-0.5 V to -2.0 V	Multiple electrolytes	C_2H_5OH (41.2%)	[2]

$CuAl_2O_4/CuO$	Titration/calcination	-	1 M KOH	C2H5OH (41%)	[3]
Cu_2O	Chemical reduction	-0.3 V	0.5 M $KHCO_3$	CH_4 (76.61%)	[4]
Ag/Cu_2O	Seed medium method	-1.0 V	3 M KCl	C_2H_4 (66%)	[5]
Cu/Cu_2O@N doped graphene	Calcination and electroreduction	-1.9 V	0.2 M KI	C2-C3 product (56%)	[6]
Cu/Cu_2O	Electrodeposition	-1.2 V	1 M KOH	C_2H_4 (57.9%)	This work

FE calculation:

Gas chromatography:

FEs for gaseous product = $e_{output}/e_{input} \times 100$

$e_{output} = (n \times V \times P)/(10^6 \times R \times T)$

$e_{input} = (I \times t)/F$

where,

e_{input} = Total number of moles of electrons measured during the sampling period.

e_{output} = Number of moles of electrons required to reduce CO_2 into gaseous products.

n = Number of electrons required to produce the products during reactions (for CO, CH_4, and C_2H_4 are 2, 8, and 12 electrons)

V= Volume of gaseous product obtained from the GC in ppm

P= Pressure at room temperature (101325 Pa)

R = Universal gas constant (8.314J/molK)

T = Room temperature in Kelvin

I = current in Ampere

t = time required to fill the 1cm^3 sample loop (1*60/flow rate)

F = Faraday's constant (96485)

^{1}H-Nuclear Magnetic Resonance:

$FE = (Q_{product}/Q_{total}) \times 100\%$

$Q_{product} = N_{product} \times n \times F$

Q_{total} = Current (A)

$N_{product}$ = The concentration of liquid product

n = Number of electrons required to make a liquid product

F = Faraday's constant (96485)

References:

(1) Shao, L.; Hu, B.; Hao, J.; Jin, J.; Shi, W.; Chen, M. A Dendritic Cu/Cu_2O Structure with High Curvature Enables Rapid and Efficient Reduction of Carbon Dioxide to C2 in an H-Cell. *Chinese J. Catal.* **2024**, *63*, 144–153.

(2) Kim, C.; Cho, K. M.; Park, K.; Kim, J. Y.; Yun, G. T.; Toma, F. M.; Gereige, I.; Jung, H. T. Cu/Cu_2O Interconnected Porous Aerogel Catalyst for Highly Productive Electrosynthesis of Ethanol from CO_2. *Adv. Funct. Mater.* **2021**, *31* (32), 2102142.

(3) Zhang, T.; Yuan, B.; Wang, W.; He, J.; Xiang, X. Tailoring *H Intermediate Coverage on the $CuAl_2O_4$/CuO Catalyst for Enhanced Electrocatalytic CO_2 Reduction to Ethanol. *Angew. Chemie - Int. Ed.* **2023**, *62* (29), e202302096.

(4) Dahal, R.; Ray, S. K.; Pathiraja, G.; Bastakoti, B. P. Low-Temperature Fabrication of Morphology-Controllable Cu_2O for Electrochemical CO_2 Reduction. *J. Mater. Sci.* **2024**, *59*, 13896.

(5) Wei, Z.; Wang, W.; Shao, T.; Yang, S.; Liu, C.; Si, D.; Cao, R.; Cao, M. Constructing Ag/Cu_2O Interface for Efficient Neutral CO_2 Electroreduction to C_2H_4. *Angew. Chemie Int. Ed.* **2025**, *64* (4), e202417066.

(6) Zhi, W.-Y.; Liu, Y.-T.; Shan, S.-L.; Jiang, C.-J.; Wang, H.; Lu, J.-X. Efficient Electroreduction of CO_2 to C2-C3 Products on Cu/Cu_2O@N-Doped Graphene. *J. CO_2 Util.* **2021**, *50*, 101594.
